# O-04 - CAN WEB SEARCHES AND PCR TESTS PREDICT HOSPITAL ADMISSIONS? INDICATOR-BASED SHORT-TERM FORECASTING OF HOSPITAL ADMISSIONS WITH ACUTE RESPIRATORY INFECTIONS IN WALES

**DOI:** 10.1093/pubmed/fdag046.004

**Published:** 2026-07-09

**Authors:** Sophie-Berenice Wilmes, Dr Warren Tennant, Drew Turner, Phillip Butterick, Rubesh Chandrasekar, Simon Cottrell, Hannah Jones, Panoraia Kalapotharakou, Mari Morgan, Emily Steggall, Christopher Williams, Sarah C Woodhall, Jiao Song

**Affiliations:** Public Health Wales; Public Health Wales; Public Health Wales; Public Health Wales; Public Health Wales; Public Health Wales; Public Health Wales; Public Health Wales; Public Health Wales; Public Health Wales; Public Health Wales; Public Health Wales; Public Health Wales

## Abstract

**Background:**

Acute respiratory infections (ARI) including influenza, RSV and COVID-19 place significant winter pressures on healthcare systems. To complement surveillance and inform winter planning by system partners, Public Health Wales developed a multi-model framework producing 14-day forecasts of ARI-related hospital admissions for 2025/26. A key component of the overall ensemble approach is a set of indicator models, which aim to capture early behavioural and surveillance signals, enabling earlier detection of shifts in infection dynamics.

**Objectives:**

To develop, implement and evaluate a suite of indicator-based models to contribute to short-term forecasting of hospital admissions with ARI in Wales.

**Methods:**

We developed a set of indicator models using real-time data from web searches (Google Trends and NHSnet website visits) and Wales-wide PCR testing data. Each indicator is incorporated through lagged predictors spanning 1–28 days. For each indicator, separate lag-specific models were fitted to hospital admissions data and evaluated on their ability to reproduce observed patterns. Forecasts were generated from all fitted models and combined using performance-based weights to produce a single daily estimate for each indicator model. We evaluated the performance of the indicator models over the 2025/26 season using a range of metrics (root-mean square error, bias and Weighted Interval Score).

**Results:**

The models were generally able to accurately predict hospital admissions, but some data availability issues were encountered during live forecasting. We identified periods where performance was reduced, including start of the flu season. We highlight potential drivers of these deviations, e.g. media publications affecting NHSnet website visits and Google Trends models.

**Conclusions:**

Overall, our results highlight the feasibility of indicator-driven models as a component of short-term forecasting of hospital admissions. These models provide a timely and flexible complement to routine ARI surveillance in Wales. Next steps include further evaluation of the utility of forecasts for end-users.

